# Antibiotics Efficiency in the Infection Complications Prevention after Third Molar Extraction: A Systematic Review

**DOI:** 10.3390/dj10040072

**Published:** 2022-04-18

**Authors:** Diana Sologova, Ekaterina Diachkova, Ilana Gor, Susanna Sologova, Ekaterina Grigorevskikh, Liana Arazashvili, Pavel Petruk, Svetlana Tarasenko

**Affiliations:** 1 Department of Oral Surgery of the Institute of Dentistry, I.M. Sechenov First Moscow State Medical University (Sechenov University), 119048 Moscow, Russia; dyachkova_e_yu_1@staff.sechenov.ru (E.D.); gor_i_a@staff.sechenov.ru (I.G.); tarasenko_s_v@staff.sechenov.ru (S.T.); 2 Department of Pharmacology, Nelyubin Institute of Pharmacy, I.M. Sechenov First Moscow State Medical University (Sechenov University), 119991 Moscow, Russia; sologova_s_s@staff.sechenov.ru (S.S.); grigorevskikh_e_m@staff.sechenov.ru (E.G.); 3 Maxillofacial Surgery Department, I.M. Sechenov First Moscow State Medical University (Sechenov University), 8-2 Trubetskaya Str., 119991 Moscow, Russia; arazashvili_l_d@staff.sechenov.ru (L.A.); petruk_p_s@staff.sechenov.ru (P.P.)

**Keywords:** third molar, extraction, antibiotic, prophylaxis, systematic review

## Abstract

(1) Background: Antibiotics are used in every medical field including dentistry, where they are used for the prevention of postoperative complications in routine clinical practice during the third molar extraction. (2) Methods: This study is reported in accordance with the Preferred Reporting Items for Systematic Reviews and Meta-Analyses (PRISMA). The present systematic review aimed to evaluate and systematize the use of antibacterial drugs in order to prevent postoperative complications in outpatient oral surgery for wisdom teeth extraction. We conducted a systematic review using electronic databases such as Medline PubMed, Scopus, and the Cochrane Central Register of Controlled Trials. Considering inclusion and exclusion criteria, we included randomized clinical trials published up to 2021 investigating the antibiotic prescription for third molar extraction. (3) Results: We selected 10 studies after the application of inclusion and exclusion criteria. The results showed that the most widely used antibiotic was amoxicillin both with and without clavulanic acid, in different dosages and duration. There were no statistically significant differences between treatment groups for development of postoperative complications. (4) Conclusions: Based on the analysis of the included studies, penicillin is currently the most widely prescribed group of antibiotics. The widespread use of this antibiotic group can lead to antimicrobial resistance (AMR). Due to increasing prevalence of bacteria resistance to penicillins, clinicians should carefully prescribe these antibiotics and be aware that the widespread use of amoxicillin may do more harm than good for the population.

## 1. Introduction

Nowadays, antibiotics are used in every medical field including dentistry. There is currently a lot of controversy on the use of this group of medicine. The more widespread the use of antibiotics becomes, the more the resistance of bacteria to antibiotics and the adverse effects associated with their use grows [1]. Antimicrobial resistance is now one of the most serious global threats to health. As a result of overuse of antibiotics, bacteria develop various resistance mechanisms to antibiotics, thereby becoming resistant to their effects. Reports from the Global Antimicrobial Surveillance System (GMS) show the occurrence of antimicrobial resistance (AMR) among 500,000 people in 22 countries [2]. In the United States, 2 million people get an AMR infection and 23,000 people die each year [3].

### Prescribing Antibiotics in Dentistry

The use of antibiotics for dental procedures has become a common practice among dentists, especially in surgery. They are indicated for treatment of odontogenic infections after teeth extractions and dental implants [4].

Extraction of the third molar is one of the most common operations in oral surgery. Sometimes, complex wisdom tooth extractions can lead to postoperative complications; therefore, antibiotic therapy is necessary. Possible side effects and antimicrobial resistance must be taken into account. The literature reports a complication rate of 4.6–30.9% after extraction of third molars [5]. Lee et al. in a 2015 study found 9.2% of complications after wisdom tooth extraction, of which severe pain comprised 4.8%, swelling 2.6%, bleeding 2.4%, alveolar osteitis 0.9%, paresthesia 0.9%, and trismus 0.5% [6]. The most frequent complications of wisdom tooth extraction are infection and dry socket. One of the rarest complications after third molars extraction is Lemierre’s syndrome which clinicians should remember [7]. Lemierre’s syndrome is a thrombophlebitis infection of the internal jugular vein and can lead to serious systemic complications such as bacteremia in the blood. Systemic antibiotics are widely used for the prevention of complications, but there is a lot of controversy for the routine prescription of antibiotics after the extraction of third molars [4].

Nowadays, electronic databases contain a large number of studies on antibiotic therapy after wisdom teeth extraction [8,9,10,11,12]. The unjustified prescription of antibiotics has serious consequences. One of these consequences is antimicrobial resistance [9,13,14,15]. It occurs when a microbe evolves to become more or completely resistant to the antimicrobial drugs with which it could previously be treated. Equally serious are side effects after the use of antibiotics such as allergic reactions to the penicillin group, which is most commonly used in dental surgery. Side effects arise due to an incomplete medical history of the patient [10,11]. As we know, antibiotics have strict indications for use but in dental surgery, clinicians prescribe antibacterial drugs prophylactically almost after each wisdom tooth extraction. Before the prescription of antibiotics, clinicians should assess general patient conditions. Systematic reviews and meta-analyses do not support the routine prescription of antibiotics after each wisdom tooth extraction [8,9,11]. In one of the latest systematic reviews, the authors made the conclusion that due to increasing antimicrobial resistance to antibiotics, clinicians should carefully administer antibiotics and remember that out of twelve patients, antibiotics can prevent only one infection [16].

When clinicians should prescribe drugs and what protocols should they use? One of the most debated issues on this topic is the need for indications for antibiotic prophylaxis and the existence of clear protocols for antibiotic use. In our study, we tried to gather as much research on the subject as possible and compile existing antibiotic prophylaxis protocols for third molar extractions.

The present systematic review aimed to evaluate and systematize the use of antibacterial drugs in outpatient oral surgery for wisdom teeth extraction in order to prevent postoperative complications.

## 2. Materials and Methods

This study was written in accordance with the recommendations of Preferred Reporting Items for Systematic Reviews and Meta-Analyses (PRISMA) statement and the Institute of Medicines’ guidelines [17].

### 2.1. Eligibility Criteria

In this systematic review, the literature search was based on the PICO (patient, intervention, comparison, and outcome) format. We selected studies that included patients of different ages and sexes who had their wisdom teeth extracted. As for the type of intervention, we included trials in which the effectiveness and comparative characteristics of antibacterial drugs were analyzed in wisdom teeth extraction. As for comparisons, we included randomized controlled trials (RCT). Regarding outcome, we excluded articles that did not research the frequency of occurrence of dry sockets and infection. The search was restricted to studies in English.

### 2.2. Sources of Information

We used 3 electronic databases which were Medline PubMed, Scopus, and the Cochrane Central Register of Controlled Trials. Studies up to 2021 were included.

### 2.3. Search Strategy

The search terms describe the PICO components: extraction; wisdom teeth, third molar; antibiotic, prophylaxis, amoxicillin; infection, dry socket.

The filters we used: full texts, humans, clinical trials, randomized controlled trials, meta-analysis, systematic review, 11 years publication date between 2011 and 2021.

The MESH strips and search algorithms were used for the search in the Medline PubMed.

Search strategy: («“antibiotic “prophylaxis” or “antibiotic” “prophylaxis” “amoxicillin”) (“third molar” “wisdom tooth”) “extraction”.

### 2.4. Selection of Studies

The date range of included articles was 2011 to 2021. Three researchers independently searched databases for the above criteria. After applying the filters, we obtained 215 results in different electronic databases. After the exclusion of articles published earlier than 2011, we obtained 92 studies. Then, we filtered 20 articles without full text and/or not written in English and received 72 results. We excluded 62 articles due to a lack of appropriate information, duplications, and the inclusion of two groups, one of which was placebo group. The final quantity of publications was 10 (Table 1).

#### Exclusion and Inclusion Criteria

Exclusion criteria:

- The articles published before 1 July 2011;
- In vitro studies or animals;
- Not full texts;
- Patients with other accompanying diseases;
- Inappropriate information in articles; or
- Articles with 2 groups, one of which was a placebo group.

Inclusion criteria:

- Randomized controlled trials (RCTs) in humans;
- Third molar extraction;
- Antibiotic prophylaxis (e.g., prevention);
- Sex: men and women;
- Age: 18+; and
- Amoxicillin use.

### 2.5. Data Collection Process and Items

The data extraction and collection process was performed twice by two independent authors in a dedicated form proposed for intervention reviews on RCTs. Disagreements were resolved through discussion.

The following data were recorded for each selected study: the source of publication (author, year, and journal of publication), the study design, participants (sample size, age, sex, and comorbidities), intervention (the name of antibiotic, dosing, and duration), and outcomes (clinical outcomes and side effects).

### 2.6. Study Risk of Bias Assessment

Risk of bias in these studies was assessed using the Cochrane “Risk of Bias tool” for randomized trials (RoB 2.0) (Higgins 2019). The Cochrane Collaboration’s tool for assessing risk of bias was used to assess the following five domains of bias in randomized controlled trials:

- The randomization process;
- Deviations from intended interventions;
- Missing outcome data;
- Measurement of the outcome; and
- Selection of the reported result.

Risk of bias was analyzed independently by two authors. We applied the tool for each study and justifications for judgements of risk of bias for each domain (low; high; some concerns). Any disagreements in the assessment of risk of bias were resolved by discussion between authors. We assessed each item (low; high; some concerns) as shown in the risk of bias table.

## 3. Results

### 3.1. Study Selection

**Figure 1:**
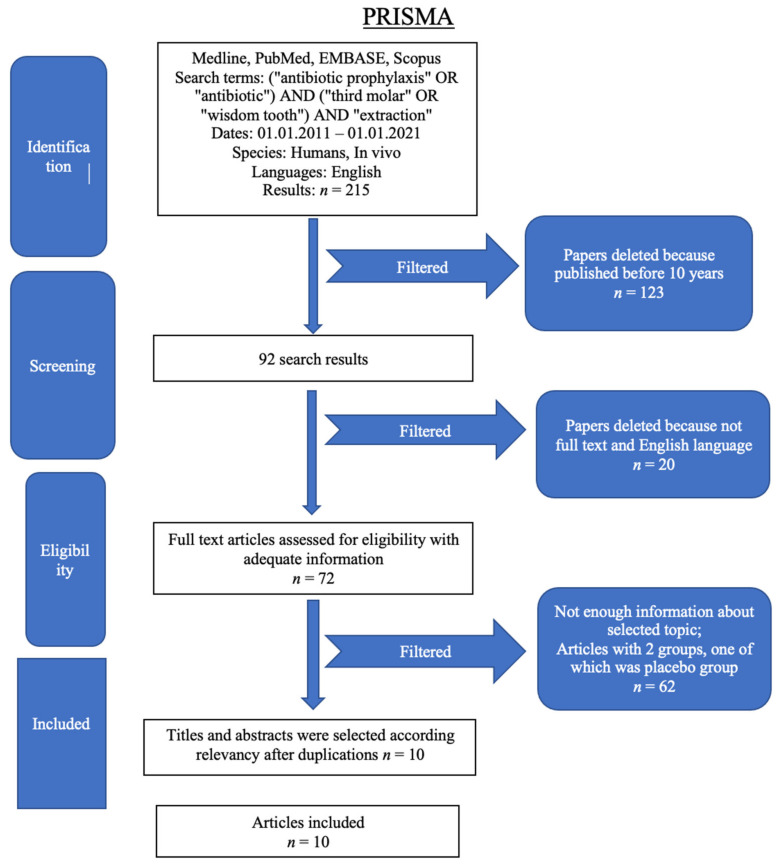
Flow diagram describing the selection process according to the Preferred Reporting Items for Systematic Reviews and Meta-Analyses (PRISMA) recommendations [17].

The study selection flow chart (Figure 1) [17] included 215 articles from databases: specifically, 175 from PubMed and 40 from Scopus databases. After elimination of articles published before 2011, 92 studies remained. Then, 20 papers were excluded because they did not have full text and/or were not written in English, leaving 72 full text articles assessed for eligibility with adequate information. Papers with not enough information and papers with two groups, one of which was placebo, were excluded. Finally, 10 papers were included in this systematic review, all of which were about use of antibiotics after third molar extraction.

### 3.2. Study Characteristics

Each of the 10 studies was RCT and included studies comprised participants without comorbidities potentially affecting their status after the surgery and not under drugs that could affect postoperative healing.

Amoxicillin with clavulanic acid was administered in three studies [20,23,25], while amoxicillin without clavulanic acid was administered in seven studies [18,19,21,22,24,26,27]. Ceftazidime and levofloxacin were administered only in one study [20,25].

The prescription of antibiotics varied in duration and dosage across studies. Antibiotics were administered only before the surgery in four studies [19,21,22,24] and only after the surgery in two studies [20,23]. In four studies, the authors compared antibiotic prescriptions in different groups before and after the surgery [18,25,26,27]. Amoxicillin was administered before and after the surgery in two studies, but the dosage of antibiotic varied [24,27].

### 3.3. Risk of Bias within Studies

Since all 10 studies included in present systematic review were randomized, risk of bias 2 (RoB2) was used (Table 2).

## 4. Discussion

The extraction of wisdom teeth in dental surgery is often accompanied by the prescription of antibiotics. However, there is little convincing evidence to support the routine use of antibiotics in the third molar extraction surgery in healthy patients [14]. Uncontrolled prescribing of antibiotics can lead to many undesirable side effects and the emergence of antibiotic-resistant micro-organisms. Nowadays, bacterial resistance to antibiotics is a serious problem. Microbial resistance to antibiotics and other drugs poses a serious threat to global health and sustainable development [29]. Therefore, many studies have discussed the question of avoiding the prescription of antibiotics after every third molar extraction. The emergence of antibiotic resistance in patients is due to the inappropriate prescribing of antibiotics. The overall health of the patient should be assessed before prescribing antibiotics to patients. Clinicians should check patients for a history of opportunistic diseases and allergies.

There are different guidelines describing the administration of antibiotics in dentistry. Italian and Belgium guidelines are similar and have strict indications of antibiotic prophylaxis use [30,31]. According to American dental association (ADA) guidelines, the prophylactic use of antibiotics should be limited to those patients who have infective endocarditis and may be at risk of developing hematogenous infections [32]. On recent recommendations, the prophylactic use of antibiotics is not recommended for patients with prosthetic joint implants [33,34].

Preoperative pain and postoperative complications can be treated by correctly used analgesic and anti-inflammatory therapy. Both cyclooxygenase inhibitors 1,2 (NSAIDs) and cyclooxygenase 3 can be used for pain management [35]. Pre and postoperative pharmacological management is complex and also includes the administration of steroid and non-steroid therapy, as it reduces the risk of postoperative inflammation complications [36]. In 2018, Weiser et al. revealed that the combination of using ibuprofen and caffeine was effective in treating acute pain after wisdom teeth extraction [35]. Karthik et al. in 2021 compared two groups of 50 people. In the study group, patients received chitosan-based microspheres incorporated with ibuprofen in the socket of the extraction tooth. In the control group, 400 mg ibuprofen tablets were administered orally following extraction of the wisdom teeth. In the study group, there was significantly less pain and better mouth opening. Chitosan-based microspheres incorporated into ibuprofen had better analgesic and anti-inflammatory properties; they reduced pain, swelling, trismus, and had reliable wound-healing properties [37]. A combination of the use of dexamethasone and etodolac was investigated in 2021 by Ramires et al. This combination of drugs led to a reduction in pain, swelling, and trismus in the postoperative period [38].

One of the causes of inflammation in the postoperative area is a high bacterial load before and after tooth extraction. In order to reduce the bacterial load, better oral hygiene and professional hygiene prior to tooth extraction is necessary. Antiseptics used immediately before the surgery can help reduce the bacterial load and therefore reduce the risk of postoperative complications. Antiseptics can be chlorhexidine in the form of a solution or as a gel. Chlorhexidine should be used for a short period of time to avoid cross-resistance to antibiotics. By observing preoperative disinfection and postoperative hygiene, the risk of infection and dry socket (DS) is reduced, thus eliminating the need to prescribe antibiotics [39,40,41,42]. Equally important during surgery is to use sterile instruments and irrigate the wound with sterile saline to reduce the bacterial load and to prevent inflammation in the postoperative area [43,44,45].

Nowadays, the most widely used antibiotic in dental surgery is amoxicillin with or without clavulanic acid [46], shown by the above-mentioned studies. Amoxicillin is used as a preferred first-line treatment due to its moderate spectrum and low adverse effects [47]. The combination of amoxicillin and clavulanic acid is used at a 7:1 ratio (875 mg amoxicillin/125 mg clavulanic acid) to avoid the toxicity of clavulanic acid such as gastrointestinal side effects [48]. Cephalosporins (for example, ceftriaxone) can be used as second-choice antibiotics instead of amoxicillin [49].

Most doctors prescribe antibiotics as a prevention against infection after extraction of a third molar, but it is necessary to differentiate postoperative pain and swelling from an infectious process. Pain and swelling persist after surgery for 48–72 h. In order to diagnose a postoperative infection and the need for antibiotics, postoperative pain and swelling must persist for more than three days and be accompanied by a fever of over 38 degrees, soft tissue inflammation, and lymphadenopathy [50]. Preoperative antibiotic prescription significantly reduces the risk of DS after tooth extraction. Prescribing antibiotics avoids one case of DS in every 13 patients. In this regard, the risk to benefit ratio must be carefully assessed because there is a risk of side effects, and antibiotic resistance (AMR) must not be forgotten [14].

### Review Study Discussion

The present systematic review, unlike previous reviews, excluded studies with two groups, one of which was a placebo group, that evaluated the difference between antibiotics or between the different dosage of the same antibiotics.

This systematic review included 10 studies that assessed the efficacy of different groups of antibiotics after third molar extraction to prevent DS, infection, and other postoperative complications. In these studies, only third molars were extracted. Pain, fever, swelling, and other postoperative complications were considered in different patient groups. The authors evaluated both local and systemic side effects after wisdom tooth extraction. Systemic side effects included headache, nausea, diarrhea, gastrointestinal reactions, and fever. Local side effects included pain, edema, bleedings, swelling, alveolar osteitis, DS, wound infection, and trismus.

Amoxicillin with or without clavulanic acid was used almost in every study. In seven studies, amoxicillin with or without clavulanic acid was used after wisdom tooth extraction and placebo was used in control group.

In the articles, the authors described results obtained both with the same antibiotic at different dosages and with different antibiotics. In most of the included studies, there were no significant statistical differences between the therapeutic groups in terms of assessed postoperative complications such as pain, edema, trismus, etc. [20,22,23,24,26]. Despite this, two papers [18,27] found significant statistical differences in postoperative complications between the therapeutic group and placebo group.

The extraction of wisdom teeth has a negative impact on postoperative quality of life due to the occurrence of postoperative complications. QOL after wisdom tooth extraction was assessed in one of the articles. Authors assessed QOL after the surgery in the amoxicillin group both pre and postoperatively, and the quality of life was much higher than in other groups [25].

Furthermore, amoxicillin group without clavulanic acid showed statistically higher presence of patients with gastrointestinal complications than amoxicillin with clavulanic acid [23].

Many clinicians overreact and immediately prescribe antibiotics to prevent the development of complications and to reduce the number of visits after the extraction of the third molar, but we should not forget the emergence of antibiotic resistance and possible side effects.

Some authors concluded that there is no advantage in routine administering antibiotics to healthy young people that undergo extraction of third molars with a controlled aseptic chain [24,26].

Routinely prescribing antibiotics for any third molar extraction can lead to adverse effects, so an individualized approach to each patient is necessary. Before prescribing antibiotics, the duration of the operation, technical difficulties, age of the patient, systemic diseases, and allergy history of the patient must be taken into account. Prescribing antibiotics without clinical justification may put patients at risk of adverse effects and contribute to the AMR [26].

Limitations: There were not many studies with other antibiotics; in almost every study, amoxicillin was prescribed, despite the fact that amoxicillin can cause allergic reactions. In most of the studies, anti-inflammatory drugs and antiseptics were compared with antibiotics. Many articles that were suitable for our inclusion and exclusion criteria were not randomized.

## 5. Conclusions

In our review, we collected the latest sources of information on the topic of antibiotic therapy for third molar extractions in which the authors highlighted trends in antibiotic prescribing practices, characterized factors contributing to the use and misuse of antibiotics in dentistry, provided insight into the importance of antibiotics, and encouraged dentists to think about prescribing antibiotics in their practice.

Based on the above-mentioned articles in our study, amoxicillin with or without clavulanic acid is currently the most commonly used antibiotic in dentistry for wisdom teeth extraction. However, the real need for antibiotic therapy is much debated in the scientific literature. Due to the current high prevalence of bacteria resistance to penicillin, the need for penicillin should be carefully assessed. The conclusion of this review is that the widely prescribed penicillins for the prevention of infections after the removal of third molars may do more harm than good for the population. Researchers are looking for ways to minimize the prescription of antibiotics in medical practice and how to differentiate clinical situations where antibiotic therapy is really needed.

## 6. Other Information

This systematic review is registered in the International prospective register of systematic reviews (PROSPERO).

The registration number: CRD42021285718.

## Figures and Tables

**Table 1:** Characteristics of included studies—randomized controlled trials.

Author	Year	Type of Study	Sample Size	Protocols	Results
López-Cedrún et al. [18]	2011	RCT	123	Preoperative amoxicillin (2 g AMX 2 h before surgery) vs. postoperative amoxicillin (15 tablets of amoxicillin 500 mg to be taken immediately after surgery 3 times daily for 5 days) vs. placebo (4 placebo tablets 2 h preoperatively and 15 tablets of placebo taken 3 times daily for 5 days)	Placebo group—5 socket infections and 1 suture dehiscence. No differences in side effects between the groups.
Siddiqi et al. [19]	2011	RCT	89	Group 1—placebo; Group 2—amoxicillin 1 g 1 h before surgery; Group 3—metronidazole 800 mg 1 h before surgery	There was no difference in surgical wound infection between groups.
Sisalli et al. [20]	2012	RCT	107	Amoxicillin clavulanate (875 mg + 125 mg per os three times a day for 5 days) vs. ceftazidime (1 g i.m., two times a day for 5 days) in postoperative period	In Group 1 (amoxicillin + clavulanic acid)—1 wound infection, 1 nausea, 1 headache, 1 diarrhea; In Group 2 (Ceftazidime)—1 heartburn
Duvall et al. [21]	2013	RCT	30	Chlorhexidine 0.12% rinse vs. amoxicillin (2 g) vs. placebo before the surgery	There were no differences between groups in bacteremia.
Bortoluzzi et al. [22]	2013	RCT	50	Group 1: 2 g of amoxicillin + 8 mg of dexamethasone; Group 2: 2 g of amoxicillin + 8 mg of placebo; Group 3: 8 mg of dexamethasone + 2 g of placebo and Group 4—placebo preoperative	1—alveolar infection, 2 cases—alveolar osteitis. No differences were between postoperative complications.
Iglesias-Martin et al. [23]	2014	RCT	546	Amoxicillin (1 g) vs. amoxicillin and clavunate (875/125 mg) postoperative	No differences between two groups. Group 1—patients with gastrointestinal complications.
Milani et al. [24]	2015	RCT	80	Amoxicillin preoperative (1 group:1 h before surgery + 500 mg 8/8 h for 7 days; 2 group:1 h before surgery + 500 mg 8/8 h for 7 days) vs. placebo preoperative (1 h before surgery and 500 mg 8/8 h for 7 days)	There was no difference between groups.
Braimah et al. [25]	2017	RCT	135	Amoxicillin with clavulanic acid (1 group: 1 gram preoperatively and then 625 mg for 5 days; 2 group: 1 g preoperatively only) vs. levofloxacin (1 g preoperatively only)	Quality of life (QoL) was assessed between 3 groups of different protocols of antibiotic treatment. There were differences between 3 groups.
Sidana et al. [26]	2017	RCT	400	Group A: anti-inflammatory drugs in the postoperative period. Group B: Amoxicillin 500 mg orally thrice daily for three days + anti-inflammatory drugs in the postoperative period only. Group C: a single dose of Amoxicillin 500 mg one hour prior to the extraction procedure. Group D: mouthwash starting 15 min prior the procedure for a period of 7 days + anti-inflammatory drugs.	There was no difference between the groups to pain, swelling, or post extraction complications.
Mariscal-Cazalla et al. [27]	2021	RCT	92	Group 1—750 mg amoxicillin before and after the surgery; Group 2 after surgery; Group 3 placebo before and after surgery.	Pain and inflammation were higher in group 3 than in groups 1 and 2.

**Table 2:** Risk of bias of randomized clinical trials, assessed through the ROB2 tool [28].

Study	The Randomization Process	Deviations from the Intended Interventions	Missing Outcome Data	Measurement of Outcome Data	Selection of the Reported Result
Sisalli et al., 2012 [20]	Some concerns	Some concerns	Low risk	Some concerns	Some concerns
Duvall et al., 2013 [21]	Low risk	Low risk	Low risk	Low risk	Low risk
Milani et al., 2015 [24]	Low risk	Low risk	Low risk	Low risk	Low risk
Braimah et al., 2017 [25]	Low risk	Low risk	Low risk	Low risk	Low risk
Iglesias-Martin et al., 2014 [23]	Some concerns	Some concerns	Low risk	Low risk	Low risk
López-Cedrún et al., 2011 [18]	Low risk	Low risk	Low risk	Low risk	Low risk
Siddiqi et al., 2011 [19]	Low risk	Low risk	Low risk	Low risk	Low risk
Sidana et al., 2017 [26]	Low risk	Low risk	Low risk	Low risk	Low risk
Bortoluzzi et al., 2013 [22]	Some concerns	Some concerns	Low risk	Low risk	Low risk
Mariscal-Cazalla et al., 2020 [27]	Low risk	Low risk	Low risk	Low risk	Low risk

## Data Availability

No new data were created in this study. Data sharing is not applicable for this study.
